# Controlling nonlinear dynamical systems into arbitrary states using machine learning

**DOI:** 10.1038/s41598-021-92244-6

**Published:** 2021-06-21

**Authors:** Alexander Haluszczynski, Christoph Räth

**Affiliations:** 1 Department of Physics, Ludwig-Maximilians-Universität, Schellingstraße 4, 80799 Munich, Germany; 2 Allianz Global Investors, risklab, Seidlstraße 24, 80335 Munich, Germany; 3 Institut für Materialphysik im Weltraum, Deutsches Zentrum für Luft- und Raumfahrt, Münchner Str. 20, 82234 Wessling, Germany

**Keywords:** Complex networks, Nonlinear phenomena

## Abstract

Controlling nonlinear dynamical systems is a central task in many different areas of science and engineering. Chaotic systems can be stabilized (or chaotified) with small perturbations, yet existing approaches either require knowledge about the underlying system equations or large data sets as they rely on phase space methods. In this work we propose a novel and fully data driven scheme relying on machine learning (ML), which generalizes control techniques of chaotic systems without requiring a mathematical model for its dynamics. Exploiting recently developed ML-based prediction capabilities, we demonstrate that nonlinear systems can be forced to stay in arbitrary dynamical target states coming from any initial state. We outline and validate our approach using the examples of the Lorenz and the Rössler system and show how these systems can very accurately be brought not only to periodic, but even to intermittent and different chaotic behavior. Having this highly flexible control scheme with little demands on the amount of required data on hand, we briefly discuss possible applications ranging from engineering to medicine.

## Introduction

The possibility to control nonlinear chaotic systems into stable states has been a remarkable discovery^1,2^. Based on the knowledge of the underlying equations, one can force the system from a chaotic state into a fixed point or periodic orbit by applying an external force. This can be achieved based on the pioneering approaches by Ott et al.^1^ or Pyragas^3^. In the former, a parameter of the system is slightly changed when it is close to an unstable periodic orbit in phase space, while the latter continuously applies a force based on time delayed feedback. There have been many extensions of those basic approaches (see e.g. Boccaletti et al.^4^ and references therein) including “anti-control” schemes^5^, that break up periodic or synchronized motion. However, all of them do not allow to control the system into well-specified, yet more complex target states such as chaotic or intermittent behavior. Further, these methods either require exact knowledge about the system, i.e. the underlying equations of motion, or rely on phase space techniques for which very long time series are necessary.

In recent years, tremendous progress has been made in the prediction of nonlinear dynamical systems by means of machine learning (ML). It has been demonstrated that not only exact short-term predictions over several Lyapunov times become possible, but also the long-term behavior of the system (its “climate”) can be reproduced with unexpected accuracy^6–12^—even for very high-dimensional systems^13–15^. While several ML techniques have successfully been applied to time series prediction, reservoir computing (RC)^16,17^ can be considered as the so far best approach, as it combines often superior performance with intrinsic advantages like smaller network size, higher robustness, fast and comparably transparent learning^18^ and the prospect of highly efficient hardware realizations^19–21^.

Combining now ML-based predictions of nonlinear systems with manipulation steps, we propose in this study a novel, fully data-driven approach for controlling nonlinear dynamical systems. In contrast to previous methods, this allows to obtain a variety of target states including periodic, intermittent and chaotic ones. Furthermore, we do not require the knowledge of the underlying equations. Instead, it is sufficient to record some history of the system that allows the machine learning method to be sufficiently trained. As previously outlined^22^, an adequate learning requires orders of magnitude less data than phase space methods.

## Results

We define the situation that requires to be controlled in the following way: A dynamical system with trajectory $\mathbf{u}$ is in state $\mathbf{X}$, which may represent e.g. periodic, intermittent or chaotic behavior. Then, the system behavior changes into another state $\mathbf{Y}$ as a consequence of order parameter changes or some uncontrollable external force. The aim of a control mechanism is now to push the system back into its original state $\mathbf{X}$, while the cause for the initial change in state is still present. This can be achieved by deriving a suitable control force $\mathbf{F} (t)$ which is applied while the system is in state $\mathbf{Y}$. Deriving $\mathbf{F} (t)$ requires the knowledge of how the trajectory $\mathbf{u} (t)$ of the system would have evolved if the system was still in state $\mathbf{X}$ instead. This ’what if’ scenario can be obtained by training a suitable machine learning technique on past observations of the system while being in state $\mathbf{X}$. In this study, this is achieved by using reservoir computing^23^, which is a recurrent neural network based approach. In principle, any other prediction method could be used instead as long as it is able to deliver good predictions. Once trained and synchronized, it can create predictions $\mathbf{v} (t)$ of arbitrary length from which the control force $\mathbf{F} (t)$ is derived as

1 $$\begin{aligned} \mathbf{F} (t)=K(\mathbf{u} (t) - \mathbf{v} (t)), \end{aligned}$$

where *K* scales the magnitude of the force. Since **F**(t) only depends on the (measured) coordinates **u**(t) and the ML prediction **v**(t), no mathematical model is required to control the system and thus the method is generally applicable as long as good predictions are available. The definition of the control force being dependent on the distance between the actual coordinate and a target coordinate is similar to what has been originally proposed by Pyragas^3^. However, in our case the control is not limited to periodic orbits but can achieve a variety of dynamical target states. A step by step description of the method is given in Section 0.2. The control of nonlinear dynamical system is studied on the example of the Lorenz system^24^, which is a model for atmospheric convection. Depending on the choice of parameters, the system exhibits e.g. periodic, intermittent or chaotic behavior. The equations read

2 $$\begin{aligned} \dot{x} = \sigma (y-x); \ \ \dot{y} = x (\rho -z)-y;\ \ \dot{z} = x y - \beta z, \end{aligned}$$

and $\varvec{\pi } \equiv (\sigma , \rho , \beta )$ are the order parameters that lead to a certain state and the trajectory is thus described by $\mathbf{u} (t)=(x(t),y(t),z(t))^{T}$. First, we simulate the Lorenz system with parameters $\varvec{\pi }$ such that we obtain the desired initial state $\mathbf{X}$. Second, we train reservoir computing on the resulting trajectory until time step $t_{train}$. Then, the parameters are shifted to $\varvec{\pi ^{*}}$ such that the system behavior changes to state $\mathbf{Y}$ at time step $t_{shift}$. If $t_{shift} \ge t_{train}$, the RC system is synchronized accordingly with the trajectory since $t_{train}$. Synchronization means that the scalar states of the reservoir (see Eq. 5) are updated but the system is not re-trained. To control the system now back into state $\mathbf{X}$, the correction force $\mathbf{F} (t)$ is derived in each time step based on the prediction $\mathbf{v} (t)$ and applied to the system by solving the differential equations of the system for the next time step including $\mathbf{F} (t)$

3 $$\begin{aligned} \mathbf{u} (t+\Delta {t}) = \int _{t}^{t+\Delta {t}} (\dot{f}(\mathbf{u} (\tilde{t}), \varvec{\pi ^{*}}) + \mathbf{F} (\tilde{t})) d\tilde{t}, \end{aligned}$$

where $\dot{f}$ is defined in Eq. (2). The knowledge of $\dot{f}$ is only required for the model system examples in this study but not for real world applications. The equations are solved using the 4th order Runge–Kutta method with a time resolution $\Delta t = 0.02$. Since still the parameters $\varvec{\pi ^{*}}$ are used, the system would continue to exhibit the undesired state $\mathbf{Y}$ if the control force was 0. For the Lorenz system, the scaling constant set to *K* = 25. We did not optimize for *K* and empirically found that our method works for a wide range of choices. It is important to emphasize that a smaller choice for K does not necessarily mean that a smaller force is needed, because smaller values may allow for more separation of **u**(t) and **v**(t).

**Figure 1:**
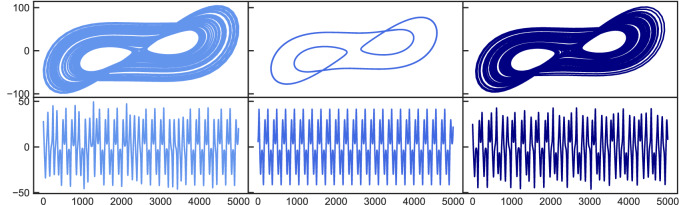
Periodic to chaotic control. Top: 2D attractor representation in the x–y plane. Bottom: X coordinate time series. Left plots show the original chaotic state which changes to a periodic state (middle) after tuning the order parameter. After applying the control mechanism, the system is forced into a chaotic state again (right).

Figure 1 shows the results for the Lorenz system originally (left side) being in a chaotic state $\mathbf{X}$ ($\varvec{\pi }=[\sigma =10.0,\rho =167.2,\beta =8/3]$), which then changes to periodic behavior (middle) $\mathbf{Y}$ after $\rho$ is changed to $\rho =166$. Then, the control mechanism is activated and the resulting attractor again resembles the original chaotic state (left). While ‘chaotification’ of periodic states has been achieved in the past, the resulting attractor generally did not correspond to a certain specified target state but just exhibited some chaotic behavior. Since we would like to not only rely on a visual assessment, we characterize the attractors using quantitative measures. First, we calculate the largest Lyapunov exponent, which quantifies the temporal complexity of the trajectory, where a positive value indicates chaotic behavior. Second, we use the correlation dimension to assess the structural complexity of the attractor. Based on the two measures, the dynamical state of the system can be sufficiently specified for our analysis. Both techniques are described in the supporting information. Because a single example is not sufficiently meaningful, we perform our analysis statistically by evaluating 100 random realizations of the system at a time. The term ’random realization’ refers to different random drawings of the reservoir $\mathbf{A}$ and the input mapping $\mathbf{W} _{in}$, as well as the initial conditions for the Lorenz system. The first line in Table 1 shows the respective statistical results for the setup shown in Figure 1. The largest Lyapunov exponent of the original chaotic system $\lambda _{orig}= 0.851$ significantly reduces to $\lambda _{changed} = 0.080$ when the parameter change drives the system into a periodic state. After the control mechanism is switched on, the value for the resulting attractor moves back to $\lambda _{controlled} = 0.0841$ and thus is within one standard deviation from its original value. Same applies to the correlation dimension, which resembles its original value after control very well.

**Table 1:** Statistical simulation over *N* = 100 random realizations of the systems evaluated in terms of the mean values of the largest Lyapunov exponent and the correlation dimension with corresponding standard deviations.

	Largest Lyapunov exponent $\lambda$			Correlation dimension $\nu$		
	$\lambda _{orig}$	$\lambda _{changed}$	$\lambda _{controlled}$	$\nu _{orig}$	$\nu _{changed}$	$\nu _{controlled}$
$Periodic \rightarrow Chaotic$	0.851 ± 0.070	0.080 ± 0.075	0.841 ± 0.074	1.700 ± 0.065	1.052 ± 0.071	1.700 ± 0.061
$Chaotic \rightarrow Intermittent$	0.571 ± 0.096	0.853 ± 0.053	0.614 ± 0.101	1.321 ± 0.086	1.678 ± 0.055	1.351 ± 0.091
$Chaotic_{B} \rightarrow Chaotic_{A}$	0.479 ± 0.060	0.643 ± 0.075	0.478 ± 0.067	1.941 ± 0.038	1.948 ± 0.047	1.933 ± 0.040
$Chaotic_{D} \rightarrow Chaotic_{C}$	0.819 ± 0.092	0.884 ± 0.058	0.822 ± 0.052	1.855 ± 0.069	1.959 ± 0.037	1.866 ± 0.050
$Periodic \leftarrow Chaotic$	- 0.003 ± 0.012	0.844 ± 0.059	0.028 ± 0.110	1.001 ± 0.065	1.700 ± 0.071	1.001 ± 0.061
$Chaotic \leftarrow Intermittent$	0.851 ± 0.070	0.550 ± 0.094	0.828 ± 0.067	1.700 ± 0.086	1.326 ± 0.055	1.698 ± 0.091
$Chaotic_{B} \leftarrow Chaotic_{A}$	0.629 ± 0.069	0.446 ± 0.068	0.629 ± 0.066	1.948 ± 0.037	1.939 ± 0.049	1.956 ± 0.037
$Chaotic_{D} \leftarrow Chaotic_{C}$	0.881 ± 0.092	0.836 ± 0.058	0.880 ± 0.052	1.958 ± 0.069	1.864 ± 0.038	1.951 ± 0.050

The subscript *orig* denotes the initial state of the system, while *changed* refers to the new state after parameters changed and *controlled* means the system controlled back into the original state. The description left to the arrow is the original state that also will be achieved again after controlling the system whereas the state written right to the arrow corresponds to the changed condition.

**Figure 2:**
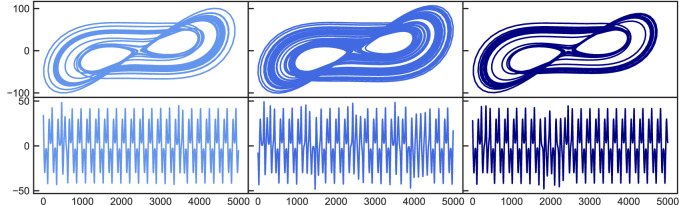
Chaotic to intermittent control. Top: 2D attractor representation in the x–y plane. Bottom: X coordinate time series. Left plots show the original intermittent state which changes to a chaotic state (middle) after tuning the order parameter. After applying the control mechanism, the system is forced into an intermittent state again (right).

Since there is a clear distinction between the chaotic- and the periodic state, with the latter being simple in terms of its dynamics, the next step is to control the system between more complex dynamics. Therefore, we start simulate the Lorenz system again with parameters $\varvec{\pi }=[\sigma =10.0,\rho =166.15,\beta =8/3]$ that lead to intermittent behavior^25^. This is shown in Fig. 2 on the left. Now $\rho$ is changed to $\rho =167.2$, which results in a chaotic state (middle plots). The control mechanism is turned on and the resulting state shows again the intermittent behavior (right plots) as in the initial state. This is particularly visible in the lower plots where only the X coordinate is shown. While the trajectory mostly follows a periodic path, it is interrupted by irregular burst that occur from time to time. It is remarkable that bursts do not seem to occur more often given the chaotic dynamics of the underlying equations and parameter setup. However, the control works so well that it exactly enforces the desired dynamics. This observation can again be confirmed by looking at the statistical results in Table 1.

**Figure 3:**
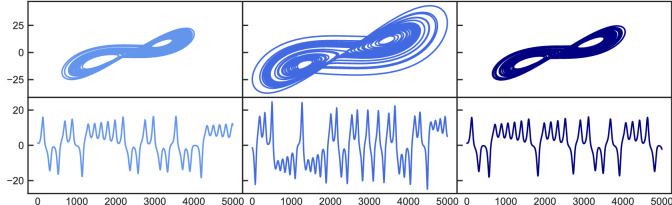
Chaotic to chaotic control. Top: 2D attractor representation in the x–y plane. Bottom: X coordinate time series. Left plots show the original chaotic state which changes to a different chaotic state (middle) after tuning the order parameter. After applying the control mechanism, the system is forced into the initial chaotic state again (right).

**Figure 4:**
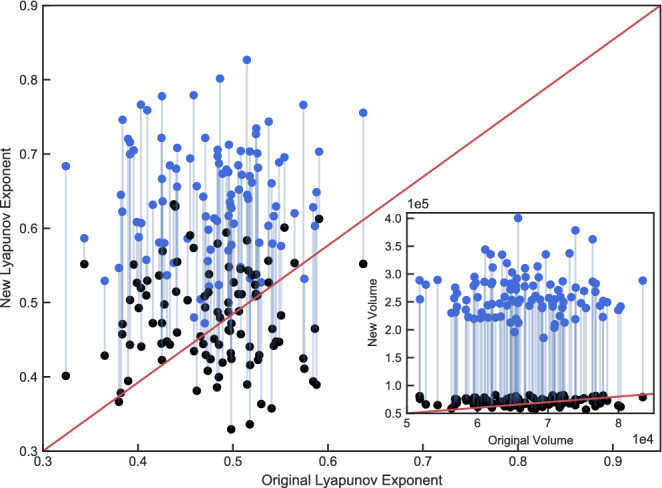
Chaotic to chaotic control ($\rho$ changed). Values on the x-axis denote the largest Lyapunov exponent $\lambda _{max}$ of the original system state before parameter change for *N* = 100 random realizations. Y-axis reflects the values for $\lambda _{max}$ after parameters changed from $\rho =28$ to $\rho =50$. The blue dots correspond to the uncontrolled systems, while the black dots represent the controlled systems. Inlay plot shows the same for the volume of the attractor.

Just like in the first two examples, it was not possible before to control a system from one chaotic state to another particular chaotic state. To do this, we start with the parameter set ($\varvec{\pi }=[\sigma =10.0,\rho =28.0,\beta =8/3]$) leading to a chaotic attractor which we call $Chaotic_{A}$. When changing $\rho$ to $rho=50.0$ we obtain a different chaotic attractor $Chaotic_{B}$. This time we use a different range of values for $\rho$ compared to the previous examples in order to present a situation where not only the chaotic dynamics change, but also the size of the attractor significantly varies between the two states. The goal of the control procedure now is to not only force the dynamics of the system back to the behavior of the initial state $Chaotic_{A}$, but also to return the attractor to its original size. Figure 3 shows that both goals succeed. This is also confirmed by the statistical results, indicating that the largest Lyapunov exponent of the controlled system is perfectly close to the one of the uncontrolled original state. For the correlation dimension, however, there are no significant deviations between the two chaotic states. To give a more striking illustration of the statistical analysis, we show the results for each of the 100 random realizations in Fig. 4. The main plot scatters the largest Lyapunov exponents as measured for the original parameter set $\varvec{\pi }$ against those measured after the parameters have been changed to $\varvec{\pi ^{*}}$. While the blue dots represent the situation where the control mechanism is not active, the control has been switched on for the black dots. Furthermore, each pair of points is connected with a line that belongs to the same random realization. It is clearly visible that the control leads to a downwards shift of the cloud of points towards the diagonal, which is consistent to the respective average values of the largest Lyapunov exponent shown in Table 1. In addition, the inlay plot shows the same logic but for the volume of the attractors being measured in terms of the smallest cuboid that covers the attractor. The control mechanism consistently works for every single realization and reduces the volume of the attractor back towards the initially desired state. We successfully applied our approach to other examples of controlling a chaotic state to another chaotic state, e.g. by varying the parameter $\sigma$ as shown in the supporting information.

The bottom half of Table 1 proves that our statements are also valid if one reverses the direction in the examples. For example, $Periodic \rightarrow Chaotic$ in the upper half of the table means, that an initially chaotic system changed into a periodic state and then gets controlled back into its initial chaotic state. In contrast, $Periodic \leftarrow Chaotic$ in the lower half now means that the system initially is in the periodic state. It then shows chaotic behavior after the parameter change and finally is controlled back into the original periodic state—thus the opposite direction as above. It is evident that all examples also succeed in the opposite direction. This supports our claim that the prediction based control mechanism works for arbitrary states.

**Figure 5:**
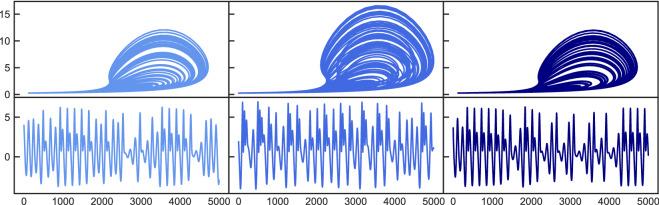
Chaotic to chaotic control for the Roessler system. Top: 2D attractor representation in the x–z plane. Bottom: X coordinate time series. Left plots show the original chaotic state which changes to a different chaotic state (middle) after tuning the order parameter. After applying the control mechanism, the system is forced into the initial chaotic state again (right).

In addition to the Lorenz system we also applied the method to another popular chaotic attractor: the Roessler system^26^. The equations read

4 $$\begin{aligned} \dot{x} = -(y+z); \ \ \dot{y} = x +ay; \ \ \dot{z} = b+(x-c)z \ \end{aligned}$$

and we use parameters $\varvec{\pi }=[a=0.5,b=2.0,c=4.0]$ leading to a chaotic behavior. This serves as our initial state and the dynamics change to another chaotic state after the parameters are changed to $\varvec{\pi ^{*}}=[a=0.55,b=2.0,c=4.0]$. For the Roessler system, we use a time resolution of $\Delta t = 0.05$ and *K* = 20. It can be seen in Fig. 5 that the control mechanism is successful. Again, the left plots represent the initial attractor resulting from the parameter set $\varvec{\pi }$. Switching to $\varvec{\pi ^{*}}$ (middle plots) not only increases the size of the attractor in the x–z plane, but also significantly changes the pattern of the x-coordinate time series. Both, the appearance of the attractor and its x-coordinate pattern become similar to the initial attractor again after the control mechanism is active (right plots). The initial state with parameters $\varvec{\pi }$ has properties $[\lambda _{max}=0.13, \nu = 1.59]$, which become $[\lambda _{max}=0.14, \nu = 1.75]$ after parameters have been changed to $\varvec{\pi ^{*}}$. Turning on the control mechanism leads to $[\lambda _{max}=0.12, \nu = 1.64]$.

## Discussion

Our method has a wide range of potential applications in various areas. For example, in nonlinear technical systems such as rocket engines it can be used to prevent the engine from critical combustion instabilities^27,28^. This could be achieved by detecting them based on the reservoir computing predictions (or any other suitable ML technique) and subsequently controlling the system into a more stable state. Here, the control force can be applied to the engine via its pressure valves. Another example would be medical devices such as pacemakers. The heart of a healthy human does not beat in a purely periodic fashion but rather shows features being typical for chaotic systems like multifractality^29^ that vary significantly among individuals. While pacing protocols developed so far aim at keeping the diastolic interval constant^30^, our general control scheme will emulate the patient-specific full behavior of the heart in healthy conditions. The control scheme could therefore be used to develop personalized pacemakers that do not just stabilize the heartbeat to periodic behavior^31–33^, but may rather adjust the heartbeat to the individual needs of the patients.

In conclusion, our machine learning enhanced method allows for an unprecedented flexible control of dynamical systems and has thus the potential to extend the range of applications of chaos inspired control schemes to a plethora of new real-world problems.

## Methods

### Reservoir computing

RC or echo state networks^17,34,35^ is an artificial recurrent neural network based approach, which builds on a static internal network called *reservoir* $\mathbf{A}$. Static means that the nodes and edges are kept fixed once the network has been initially created. This property makes RC computationally very efficient, as only its linear output layer is being optimized in the training process. The reservoir $\mathbf{A}$ is constructed as a sparse Erdös–Renyi random network^36^ with $D_{r}=300$ nodes that are connected with a probability *p* = 0.02. In order to feed the *D* = 3 dimensional input data $\mathbf{u} (t)$ into the reservoir $\mathbf{A}$, we set up an $D_{r} \times D$ input mapping matrix $\mathbf{W} _{in}$, which defines how strongly each input dimension influences every single node. The dynamics of the network are represented by its $D_{r} \times 1$ dimensional scalar states $\mathbf{r} (t)$ evolving according to the recurrent equation

5 $$\begin{aligned} \mathbf{r} (t+ \Delta {t}) = tanh(\mathbf{A} {} \mathbf{r} (t) + \mathbf{W} _{in} \mathbf{u} (t)). \end{aligned}$$

Output $\mathbf{v} (t + \Delta {t})$ is created by mapping back $\mathbf{r} (t)$ using a linear output function $\mathbf{W} _{out}$ such that

6 $$\begin{aligned} \mathbf{v} (t) = \mathbf{W} _{out}(\tilde{\mathbf {r}}(t), \mathbf{P} ) = \mathbf{P} \tilde{\mathbf {r}}(t), \end{aligned}$$

where $\tilde{\mathbf {r}} = \{\mathbf {r}, \mathbf {r}^{2}\}$. The matrix $\mathbf{P}$ is determined in the training process. This is done by acquiring a sufficient number of reservoir states $\mathbf{r} (t_{w}\ldots t_{w}+t_{T})$ and then choosing $\mathbf{P}$ such that the output $\mathbf{v}$ of the reservoir is as close as possible to the known real data $\mathbf{v} (t_{w}\ldots t_{w}+t_{T})$. For this we use Ridge regression, which minimizes

7 $$\begin{aligned} \sum ^{}_{-T \le t \le 0} {\parallel \mathbf{W} _{out}(\tilde{\mathbf {r}}(t), \mathbf{P} ) - \mathbf{v} _{R}(t) \parallel }^2 - \beta {\parallel \mathbf{P} \parallel }^2, \end{aligned}$$

where $\beta$ is the regularization constant that prevents from overfitting by penalizing large values of the fitting parameters. The training process only involves the linear output layer and therefore is fast compared to other ML methods. Replacing $\mathbf{u} (t)$ in the $\textit{tanh}$ activation function above by $\mathbf{P} \tilde{\mathbf {r}}(t)$ allows to create predictions of arbitrary length due to the recursive equation for the reservoir states $\mathbf{r} (t)$:

8 $$\begin{aligned} \mathbf{r} (t+ \Delta {t})&= tanh(\mathbf{A} {} \mathbf{r} (t) + \mathbf{W} _{in} \mathbf{W} _{out}(\tilde{\mathbf {r}}(t),\mathbf{P} )) \nonumber \\&= tanh(\mathbf{A} {} \mathbf{r} (t) + \mathbf{W} _{in} \mathbf{P} \tilde{\mathbf {r}}(t)). \end{aligned}$$

Further details including the choices for the hyperparameters are presented in the supporting information. We use a washout phase of 1000 time steps, a training period of 5000 time steps and let the parameter change of the dynamical system from $\varvec{\pi }$ to $\varvec{\pi ^{*}}$ happen immediately after the training period and thus the prediction is needed from this moment on. However, it is not necessary that the network is trained on the full history until the parameter change happened. In general, it needs to be sufficiently trained and can then be synchronized based on the recorded trajectory after the training ended. The prediction is carried out for 10,000 time steps.

It has been shown by Bompas et al.^18^ that the performance of reservoir computing does not strongly depend on the precision of the data. Hence, measurement noise and sensitive dependence on initial conditions for chaotic systems is not a problem when it comes to real world applications of the proposed method.

### Control mechanism

The concrete steps of the application of the control mechanism to the examples in our study are shown in Algorithm 1. This is the simplest setup possible, where only one long prediction for **v**(t) is performed before the control force is activated. We also successfully tested multiple more complicated setups, e.g. where the control force is not immediately switched on and the system is running on the new parameters $\varvec{\pi ^{*}}$ (and thus state $\mathbf{Y}$) for a while, where the reservoir computing prediction is updated after synchronizing the RC model with the realized trajectory since the last training or where the force is not applied in every time step. The control phase is run for 10,000 time steps.

These steps also apply for real world systems, where no mathematical model is available. The only requirement is sufficient data of the system recorded while being in the desired dynamical state $\mathbf{X}$.

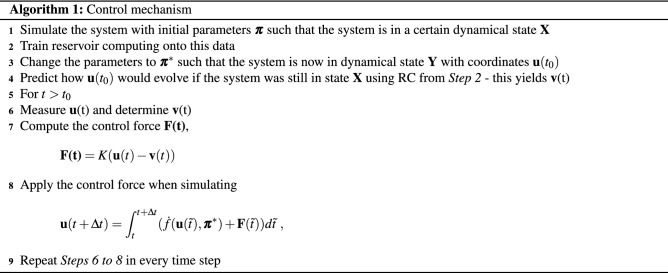



### Correlation dimension

To characterize the attractor and therefore its dynamical state we rely on quantitative measures. For this, we are looking at the long-term properties of the attractor rather than its short-term trajectory. One important aspect of the long-term behavior is the structural complexity. This can be assessed by calculating the correlation dimension of the attractor, where we measure the dimensionality of the space populated by the trajectory^37^. The correlation dimension is based on the correlation integral

9 $$\begin{aligned} C(r)&= \lim \limits _{N \rightarrow \infty }{\frac{1}{N^2}\sum ^{N}_{i,j=1}\theta (r- | \mathbf{x} _{i} - \mathbf{x} _{j} |)} \nonumber \\&= \int _{0}^{r} d^3 r^{\prime } c(\mathbf{r} ^{\prime }), \end{aligned}$$

where $\theta$ is the Heaviside function and $c(\mathbf{r} ^{\prime })$ denotes the standard correlation function. The correlation integral represents the mean probability that two states in phase space are close to each other at different time steps. This is the case if the distance between the two states is less than the threshold distance *r*. The correlation dimension $\nu$ is then defined by the power-law relationship

10 $$\begin{aligned} C(r) \propto r^{\nu }. \end{aligned}$$

For self-similar strange attractors, this relationship holds for a certain range of *r*, which therefore needs to be properly calibrated. As we are finally only interested in comparisons, precision with regards to absolute values is not essential here. We use the Grassberger Procaccia algorithm^38^ to calculate the correlation dimension.

### Lypunov exponents

The temporal complexity of a system can be measured by its Lyapunov exponents $\lambda _{i}$, which describe the average rate of divergence of nearby points in phase space, and thus measure sensitivity to initial conditions. There is one exponent for each dimension in phase space. If the system exhibits at least one positive Lyapunov exponent, it is classified as chaotic. The magnitudes of $\lambda _{i}$ quantify the time scale on which the system becomes unpredictable^39,40^. Since at least one positive exponent is the requirement for being classified as chaotic, it is sufficient for our analysis to calculate only the largest Lyapunov exponent $\lambda _{max}$

11 $$\begin{aligned} d(t) = C e^{\lambda _{max} t}. \end{aligned}$$

This makes the task computationally much easier than determining the full Lyapunov spectrum. We use the Rosenstein algorithm^41^ to obtain it. In essence, we track the distance *d*(*t*) of two initially nearby states in phase space. The constant *C* normalizes the initial separation. As for the correlation dimension, we are interested in a relative comparison that characterizes states of the system rather than the exact absolute values. It is important to point out that both measures—the correlation dimension and the largest Lyapunov exponent—are calculated purely based on data and do not require any knowledge of the underlying equations.

## Supplementary information

Supplementary Information.

## Data Availability

The data that support the findings of this study are available from the corresponding author upon reasonable request.
