# Supplementary material for: Controlling nonlinear dynamical systems into arbitrary states using machine learning
Source: Sci Rep. 2021 Jun 21;11:12991. doi: 10.1038/s41598-021-92244-6 (PMC8217470; doi:10.1038/s41598-021-92244-6)
Supplement: Supplementary file 1 — Supplementary Information. [file 41598_2021_92244_MOESM1_ESM.pdf]

Supplementary Material

Reservoir Computing

For the control mechanism to work it is crucial that good predictions of the system are available. In the study, we present the Lorenz system as an example of a complex nonlinear system which is controlled into different states. In order to obtain a good prediction of such a system, we use reservoir computing (RC)[1–3]. RC is an artificial recurrent neural network based approach, which relies on a static internal network called *reservoir*. Static means that the nodes and edges are kept fixed once the network has been initially constructed. This property makes RC computationally very efficient, as only its linear output layer is being optimized in the training process. Thus, even high model dimensionality is computationally feasible, which makes the model well suited for complex real-world applications.

The implementation is mainly based on the setup of our previous study[4]. The reservoir $\mathbf{A}$ is constructed as a sparse Erdös-Renyi random network[5] with dimensionality D_r. We chose to connect $D_r = 300$ nodes that are connected with a probability $p = 0.02$, such that we get an average unweighted degree of $d = 6$. Initially, the weights of the edges are determined by independently drawn and uniformly distributed random numbers within the interval $[-1, 1]$. A system parameter of particular interest is the spectral radius ρ of the reservoir $\mathbf{A}$, which is defined as its largest absolute eigenvalue

$$\rho(\mathbf{A}) = max\{|\lambda_1|, ..., |\lambda_{D_r}|\} \ , \tag{1}$$

and can be interpreted as the average degree of the network. Given a fixed size D_r of the reservoir, the magnitude of ρ then depends on the number of edges and their weights. Therefore, one can adjust it as follows:

$$\mathbf{A}^* = \frac{\mathbf{A}}{\rho(\mathbf{A})}\rho^* \ . \tag{2}$$

Here, ρ^* is the desired spectral radius. In order to feed the D dimensional input data $\mathbf{u}(t)$ into the reservoir $\mathbf{A}$, we set up an $D_r \times D$ input mapping matrix $\mathbf{W}_{in}$, which defines how strongly each input dimension influences every single node. The entries of $\mathbf{W}_{in}$ are chosen to be uniformly distributed random numbers within the interval $[-\omega, \omega]$. We will specify ω and ρ^* in a second. The dynamics of the network are represented by its $D_r \times 1$ dimensional scalar states $\mathbf{r}(t)$. Being initially set to $r_i(t_0) = 0$ for all nodes, they evolve according to the recurrent equation

$$\mathbf{r}(t + \Delta t) = \alpha \mathbf{r}(t) + (1 - \alpha) tanh(\mathbf{A}\mathbf{r}(t) + \mathbf{W}_{in}\mathbf{u}(t)) \ . \tag{3}$$

In this study we set $\alpha = 0$, and thus do not mix the input function (argument of the *tanh*, which is a function of $\mathbf{r}(t)$) with past reservoir states $\mathbf{r}(t)$ directly. In order to achieve optimal predictions, the choice for the scaling ω of the input function and the desired spectral radius ρ^* of the reservoir is crucial. As shown in our previous study[6], the values should be chosen such that the distribution of the arguments of the hyperbolic tangent activation function lies within the dynamical range of the hyperbolic tangent function. This depends of course also on the ranges of the input data itself. If ω and ρ^* were too large, then most values would be in the saturation regime of the *tanh* function. In contrast, too small choices would lead to an approximately linear behavior. However, the nonlinearity of the activation function is an important requirement for good predictions of complex nonlinear systems. In this study we did not optimize for both parameters, but heuristically set reasonable values based on our insights from[6], which depend on the example and are summarized in Table 1. In order to get the D dimensional output $\mathbf{v}(t)$ from the reservoir states $\mathbf{r}(t)$, an output function $\mathbf{W}_{out}$ is used that linearly depends on some output mapping matrix $\mathbf{P}$

$$\mathbf{v}(t) = \mathbf{W}_{out}(\mathbf{r}(t), \mathbf{P}) = \mathbf{P}\tilde{\mathbf{r}}(t) \ , \tag{4}$$

where $\tilde{\mathbf{r}}(t)$ is a function of $\mathbf{r}(t)$. Often $\tilde{\mathbf{r}}(t) = \mathbf{r}(t)$ is used, but this leads to severe problems due to the antisymmetry of the hyperbolic tangent as explained in[7]. To break this symmetry, we choose $\tilde{\mathbf{r}} = \{\mathbf{r}, \mathbf{r}^2\}$. This means that we append the squared elements of the reservoir states $\mathbf{r}^2 = \{r_1^2, r_2^2, ..., r_{D_r}^2\}$. The output mapping matrix $\mathbf{P}$ then contains $2D_r \times D$ degrees of freedom and determining its coefficients is called *training*. This is done by acquiring a sufficient number of reservoir states $\mathbf{r}(t_w ... t_w + t_T)$ and then choosing $\mathbf{P}$ such that the output $\mathbf{v}$ of the reservoir is as close as possible to the known real data $\mathbf{v}(t_w ... t_w + t_T)$. For this we use Ridge regression, which minimizes

$$\sum_{-T \le t \le 0} \parallel \mathbf{W}_{out}(\tilde{\mathbf{r}}(t), \mathbf{P}) - \mathbf{v}_R(t) \parallel^2 - \beta \parallel \mathbf{P} \parallel^2 \ , \tag{5}$$

where β is the regularization constant that prevents from overfitting by penalizing large values of the fitting parameters. The notation $\parallel \mathbf{P} \parallel$ describes the sum of the square elements of the matrix $\mathbf{P}$. This ridge regression problem can be solved in matrix form[8] reading

$$\mathbf{P} = (\tilde{\mathbf{r}}^T \tilde{\mathbf{r}} + \beta \mathbb{1})^{-1} \tilde{\mathbf{r}}^T \mathbf{v}_R \ . \tag{6}$$

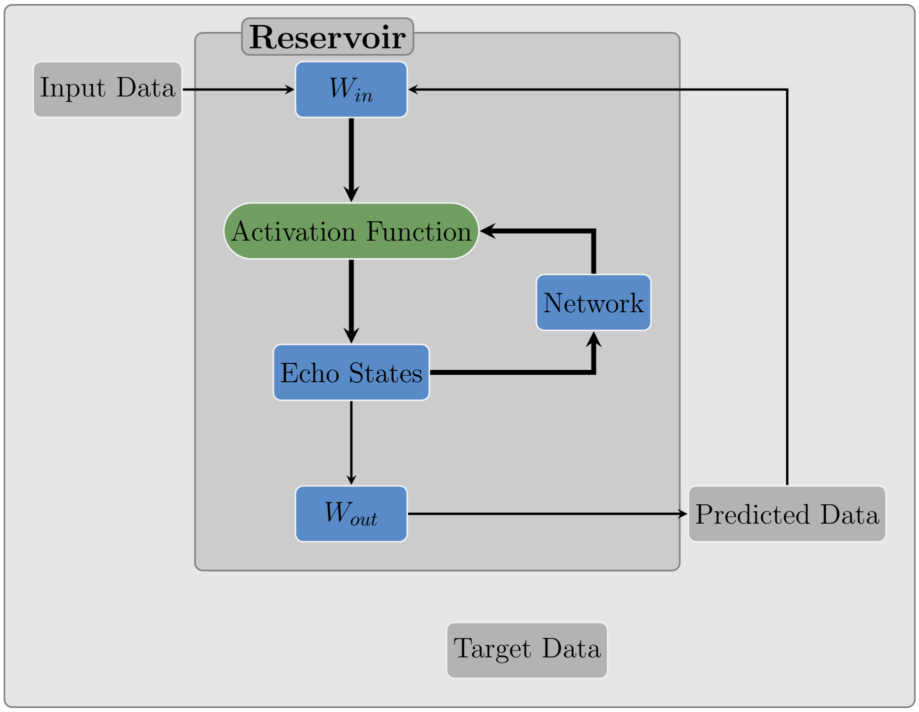

Figure 1. Schematic illustration of reservoir computing taken from[6].

The notion $\mathbf{r}$ and $\mathbf{v}_R$ without the time indexing t denotes matrices, where the columns contain the vectors $\mathbf{r}(t)$ and $\mathbf{v}_R(t)$ respectively for every time step. Before recording the reservoir states $\mathbf{r}$, the system runs through an initialization or washout phase for $t_w = 1000$ time steps in order to allow for sufficient synchronization with the dynamics of the input signal $\mathbf{u}$. After this, the RC system is trained for $t_T = 5000$ time steps.

Once $\mathbf{P}$ has been trained, the predicted state $\mathbf{v}(t)$ can be fed back in the activation function as input instead of the actual data $\mathbf{u}(t)$ by combining Eq 3 and Eq 4. This allows to create predicted trajectories of arbitrary length due to the recursive equation for the reservoir states $\mathbf{r}(t)$:

$$\mathbf{r}(t+\Delta t) = tanh(\mathbf{A}\mathbf{r}(t) + \mathbf{W}_{in}\mathbf{W}_{out}(\tilde{\mathbf{r}}(t), \mathbf{P})) \quad (7)$$

$$= tanh(\mathbf{A}\mathbf{r}(t) + \mathbf{W}_{in}\mathbf{P}\tilde{\mathbf{r}}(t)) \ . \quad (8)$$

A schematic overview of the above described reservoir computing framework is given in Fig 1.

Further Results

We presented three visual examples (plots of the attractors) for our control mechanism in the study, while statistical results are provided for further examples. In particular, we tried some more parameter sets where a chaotic state is controlled to another different chaotic state. Figure 2 shows an example where we did not vary ρ as in the other examples but σ. This corresponds to the case $Chaotic_D \rightarrow Chaotic_C$ in the study and the parameters used for the reservoir computing prediction can be found in Table 1. The initial parameters are $\boldsymbol{\pi} = [\sigma = 10.0, \rho = 102.0, \beta = 8/3]$, which lead to a chaotic behavior of the Lorenz system. Those are then changed to $\boldsymbol{\pi}^* = [\sigma = 20.0, \rho = 102, \beta = 8/3]$ leading to another chaotic state. The initial state is shown in the left plots of Fig 2, while the attractor based on the new parameters $\boldsymbol{\pi}^*$ is shown in the middle. Both attractors have approximately the same size but look different. This can particularly be observed when looking on the x coordinates only (bottom plots). We can also quantify the differences based on the measures we introduced to characterize the temporal and structural complexity of an attractor: The largest Lyapunov exponent λ_{max} and the correlation dimension ν. While the initial state has the properties $[\lambda_{max} = 0.90, \nu = 1.88]$, the second state is characterized by $[\lambda_{max} = 0.84, \nu = 1.91]$ and therefore has a slightly lower largest Lyapunov exponent with the correlation dimension being quite similar. After the control mechanism is switched on, the attractor now (right plot) looks again like the initial attractor (left plot) although the simulation still runs with the changed parameters $\boldsymbol{\pi}^*$. However, the control force F, which is based on the reservoir computing prediction that has been trained on the initial parameter set $\boldsymbol{\pi}$, pushes the system successfully back into its desired initial state. The dynamical properties

Table 1. Summary of parameters of the reservoir computing predictions used in the examples shown in the study

	ω	ρ^*	β
$Periodic \rightarrow Chaotic$	0.0084	0.0084	$6*10^{-11}$
$Chaotic \rightarrow Intermittent$	0.0084	0.0084	$1*10^{-11}$
$Chaotic_B \rightarrow Chaotic_A$	0.0100	0.0100	$1*10^{-11}$
$Chaotic_D \rightarrow Chaotic_C$	0.0150	0.0150	$1*10^{-11}$
$Periodic \leftarrow Chaotic$	0.0084	0.0084	$6*10^{-11}$
$Chaotic \leftarrow Intermittent$	0.0084	0.0084	$1*10^{-11}$
$Chaotic_B \leftarrow Chaotic_A$	0.0025	0.0025	$1*10^{-11}$
$Chaotic_D \leftarrow Chaotic_C$	0.0120	0.0120	$1*10^{-11}$
$Roessler\ Chaotic_B \rightarrow Roessler\ Chaotic_A$	0.8	0.4	$1*10^{-10}$

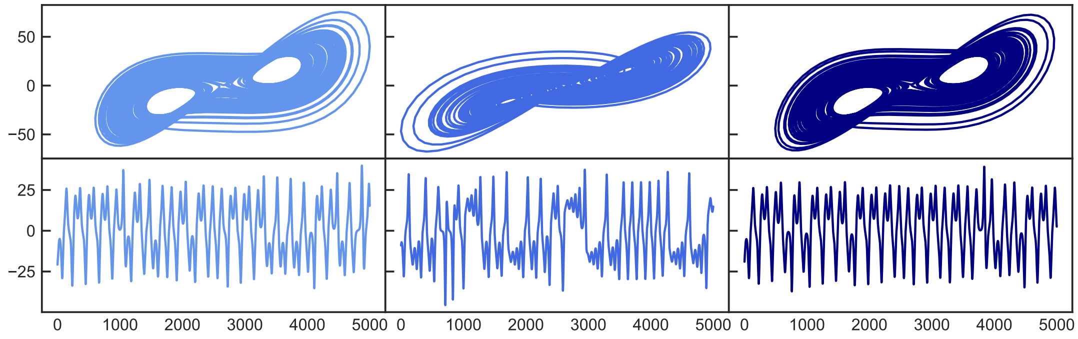

Figure 2. Chaotic to chaotic control. Top: 2D attractor representation in the x-y plane. Bottom: X coordinate time series. Left plots show the original chaotic state which changes to a different chaotic state (middle) after tuning the order parameter. After applying the control mechanism, the system is forced into the initial chaotic state again (right).

of the controlled attractor are $[\lambda_{max} = 0.90, \nu = 1.87]$ and therefore the Lyapunov exponent takes on the same value as for the original attractor. The correlation dimension stays about the same, which can be a result of the fuzziness of the calculation method used.

References

1. Jaeger, H. The "echo state" approach to analysing and training recurrent neural networks-with an erratum note. *Bonn, Ger. Ger. Natl. Res. Cent. for Inf. Technol. GMD Tech. Rep.* **148**, 13 (2001).

2. Maass, W., Natschläger, T. & Markram, H. Real-time computing without stable states: A new framework for neural computation based on perturbations. *Neural computation* **14**, 2531–2560 (2002).

3. Jaeger, H. & Haas, H. Harnessing nonlinearity: Predicting chaotic systems and saving energy in wireless communication. *science* **304**, 78–80 (2004).

4. Haluszczynski, A. & Räth, C. Good and bad predictions: Assessing and improving the replication of chaotic attractors by means of reservoir computing. *Chaos: An Interdiscip. J. Nonlinear Sci.* **29**, 103143 (2019).

5. Erdos, P. On random graphs. *Publ. mathematicae* **6**, 290–297 (1959).

6. Haluszczynski, A., Aumeier, J., Herteux, J. & Räth, C. Reducing network size and improving prediction stability of reservoir computing. *Chaos: An Interdiscip. J. Nonlinear Sci.* **30**, 063136 (2020).

7. Herteux, J. & Räth, C. Reservoir computing and its sensitivity to symmetry in the activation function. *arXiv preprint arXiv:2010.07103* (2020).

8. Hoerl, A. E. & Kennard, R. W. Ridge regression: Biased estimation for nonorthogonal problems. *Technometrics* **12**, 55–67 (1970).
